# New Mitogenomic Resources for the *Caiman yacare* (Daudin, 1802) From Bolivia

**DOI:** 10.1002/ece3.73336

**Published:** 2026-03-26

**Authors:** Guido Miranda, Ninón Ríos, Nicolas Hubert

**Affiliations:** ^1^ Wildlife Conservation Society Bolivia Program La Paz Plurinational State of Bolivia; ^2^ ISEM Univ Montpellier, CNRS, IRD Montpellier France; ^3^ Observatoire de la Biodiversité de L'Amazonie Péruvienne (OBAP—IRD, IIAP) Iquitos Peru

**Keywords:** Bolivia, *Caiman yacare*, Illumina sequencing, mitochondrial genomes, species delimitation methods

## Abstract

This study addresses the evolutionary state of a newly discovered population of *Caiman yacare* outside the western edge of its range in Bolivia by providing new mitochondrial genomic resources. Despite their conservation importance, few complete mitochondrial genomes have been generated for South American crocodilians, and most of those available originate from captive individuals, thereby limiting their utility for conservation and taxonomic applications. To bridge this gap, we generated complete mitochondrial genomes for six wild individuals from this newly discovered population in Bolivia. These data complement previously available sequences and provide essential resources for conservation and forensic applications. Mitogenomes were assembled and annotated using MEGAHIT and MitoZ. Species delimitation analyses using five independent methods revealed seven mitochondrial lineages (MOTUs) within the genus *Caiman*, while *C. yacare* itself formed a single MOTU with two phylogroups of distinct geographic distributions. All six new genomes clustered within the Madeira phylogroup. Phylogenetic and coalescent analyses suggest that this newly documented population retains high haplotypic diversity despite being located at the margin of the species range distribution. This study provides the first mitogenomes of wild *C. yacare* and contributes valuable data for understanding population structure, lineage divergence, and for informing management strategies.

## Graphical abstract

New mitochondrial genomes were generated to enhance the mitochondrial genetic resources available for Caiman yacare. Mitochondrial genomes of approximately 16 kb were obtained through genome skimming using Illumina sequencing technology. The assemblies revealed a composition typical of vertebrate mitogenomes, including 13 protein‐coding genes, 2 rRNA genes, and 22 tRNA genes. The four recovered haplotypes correspond to the Bolivian lowland phylogroup and suggest high genetic diversity within wild populations.

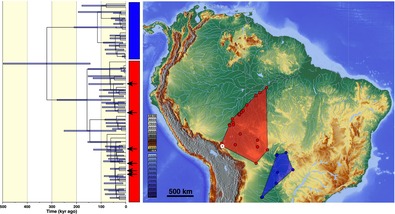

## 1 Introduction

Informative and easily accessible molecular markers are essential for the conservation of emblematic and endangered animal species. Due to its maternal inheritance and high mutation rate, the mitochondrial genome has long been a preferred source for such markers. For instance, the mitochondrial genome has been successfully used to investigate phylogeographic patterns (Bernatchez and Wilson 1998; Avise 2000) and identify species (Blaxter 2003; Hebert et al. 2003) in animals. With the development of next‐generation sequencing (NGS) methods, the ease of accessing large volumes of nuclear markers has led to a relative decline in interest in mitochondrial markers, which, due to their maternal inheritance, are more limited for examining ongoing gene flow and reproductive isolation (Formenti et al. 2022). However, the development of environmental DNA methods and the growing importance of DNA barcoding in taxonomy have led to a renewed interest in mitochondrial markers such as cytochrome c oxidase I or ribosomal RNA genes (Bohmann et al. 2014; Deiner et al. 2017; Boivin‐Delisle et al. 2021). As such, the availability of mitochondrial resources has become pivotal for the conservation of emblematic species by enabling new opportunities through mitogenomic applications such as environmental DNA.

Crocodilian species are of significant conservation concern, as many have historically been subjected to intense commercial hunting—particularly for their skins—resulting in population declines across various regions of South America (Plotkin et al. 1983; Da Silveira and Thorbjarnarson 1999; Marioni et al. 2021). Despite their importance in conservation, mitochondrial resources for South American crocodilians remain limited to a few markers (Roberto et al. 2020; Amavet et al. 2023) or to complete mitochondrial genomes from a small number of captive‐bred individuals (Pan et al. 2021), thereby restricting their applicability in biodiversity forensics and conservation. In this context, the generation of complete mitochondrial genomes for South American crocodilians is timely.

The *Caiman yacare* (Daudin, 1802) is notable for its wide distribution across the Amazon basin and the Pantanal region, as well as the potential existence of hidden lineages within the species (Roberto et al. 2020). Moreover, this species has been particularly impacted by habitat loss and poaching—two factors that may have intensified processes of population fragmentation and isolation (Campos et al. 2010). In Bolivia, recent genetic studies have identified at least three taxa within the genus *Caiman*: (1) *Caiman yacare* , found in the eastern tributaries of the Madeira River; (2) *Caiman crocodilus* (Linnaeus, 1758), known from the Madre de Dios region; and (3) *Caiman latirostris* (Daudin, 1802), restricted to the Plata Basin (Roberto et al. 2020; Amavet et al. 2023). Although there are records on the general distribution, abundance, and population status of *Caiman yacare* across broad areas of the Beni and Bolivian Pantanal (King and Videz‐Roca 1989), a significant knowledge gap remains regarding isolated populations which may represent historical relics, ecological refuges, or populations founded by occasional dispersal events.

**FIGURE 1:**
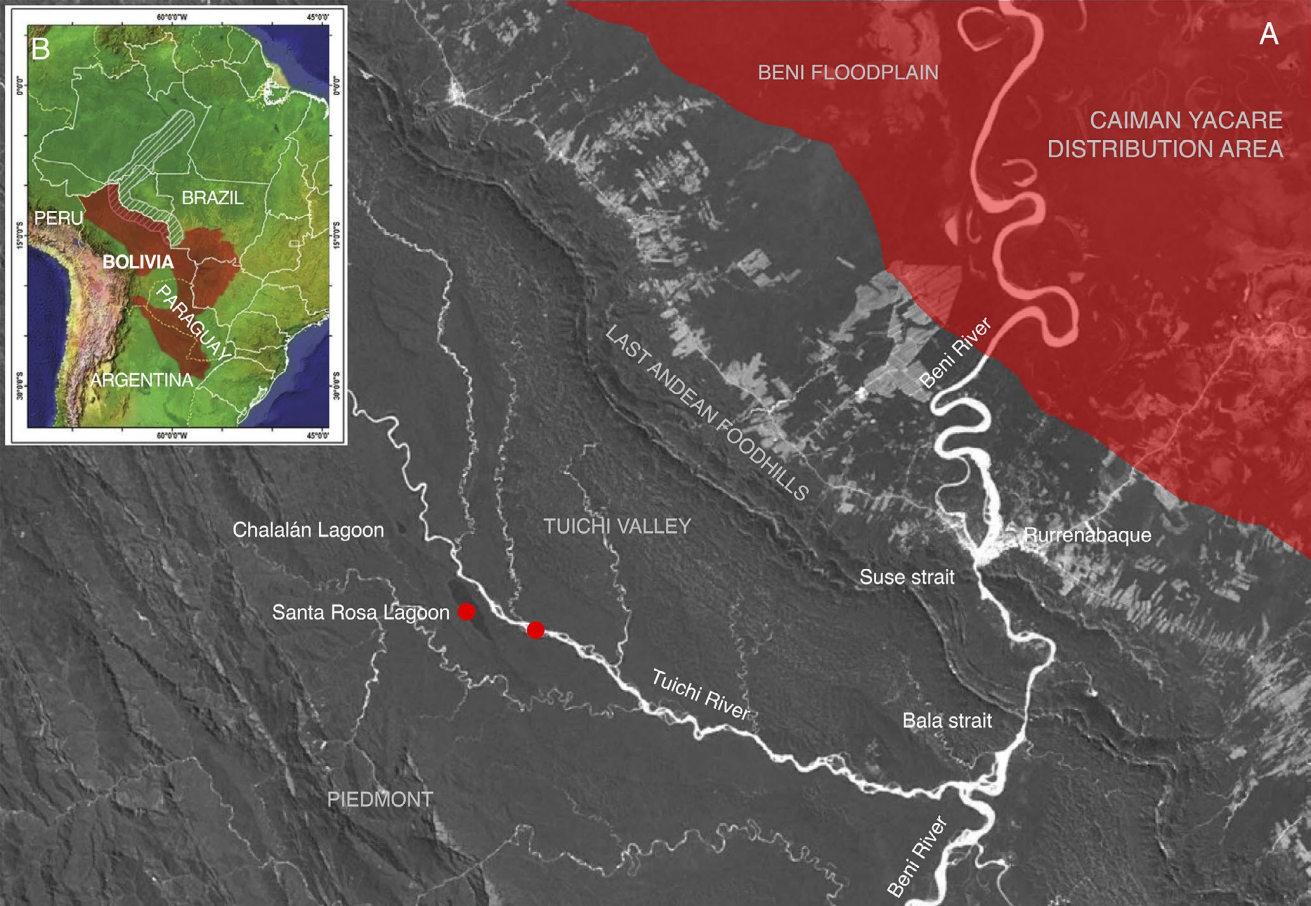
Collecting sites of the specimens of *Caiman jacare*. (A) Detail of Santa Rosa lake and its location in relation to the Tuichi River, (B) Distribution range of *Caiman yacare* according to Farias et al. (2013) and Amavet et al. (2023). Red dots represent the sampling sites and red polygon represent the known range distribution of *Caiman yacare* in the area. Continental map modified from Farias et al. (2013).

A particularly interesting case is the recent discovery of an isolated population of *C. yacare* in the Tuichi River, a tributary of the Beni River, located in Andean piedmont lagoons at approximately 330 m above sea level, a site currently outside of the known range of the species in the area (Figure 1). This newly discovered population appears to be isolated by a series of narrow channels that limit hydrological connectivity with the lower Beni River. In this context, complete mitochondrial genomes of several *C. yacare* individuals from the Tuichi River were generated with the following objectives: (1) to confirm the identity of the population using species delimitation methods, (2) to examine the maternal genetic structure of this population in comparison to lowland populations using phylogenetic methods. This information is crucial for understanding the levels of isolation, the risks associated with genetic and ecological disconnection, and the potential management needs required to ensure the conservation of this population.

## 2 Materials and Methods

### 2.1 Collecting Site

This study was conducted at Santa Rosa oxbow lake and the Tuichi River, a tributary of the Beni River, located in the tropical Andes region of Bolivia, near the town of San Buenaventura in the Department of La Paz (Figure 1). This area represents an important ecological transition between the Andean foothills and the Amazonian lowlands. The Tuichi River valley, where the caimans were sampled, lies upstream of two straits, Suse and Bala, where the Beni River crosses the last mountain ranges of the Andes. These straits are characterized by depths exceeding 45 m and represent the narrowest section of the Beni River. As shown in Figure 1, this zone is located outside the known edge of the natural distribution range of the *Caiman yacare* (Amavet et al. 2023).

### 2.2 Sampling

Individuals were manually captured using a capture noose attached to a 130 cm handle, a selective technique that allows for the safe restraint of the animal while minimizing the risk of injury to both the specimens and the researchers. Once the individual was restrained, tissue samples were collected by cutting the distal tip of scales from the caudal region, specifically at the base of the tail. The collected samples were preserved in 96% ethanol, individually labeled, recording the date, locality, sex (when possible, to determine), and estimated body size. Each individual was handled following animal welfare protocols, keeping the immobilization time as short as possible and releasing the animals immediately after sample collection. Sampling was authorized through Scientific Collection Permit No. MMAYA‐VMABCCGDF‐DGBAP‐UGCE‐0282‐CAR/24, issued by the General Directorate of Biodiversity and Protected Areas (DGBAP) of the Ministry of Environment and Water of Bolivia. Additionally, for the transport and export of biological samples intended for analyses outside the country, the respective export permits were obtained under Export Permit No. CAR/MMAYA/V/MABCCGDF/DGBAP/MEG NO 0419/2022, also granted by the DGBAP. Both the collection and export procedures complied with national and international regulations, including the provisions of the Convention on International Trade in Endangered Species of Wild Fauna and Flora (CITES), given the regulated status of the species.

### 2.3 Genomic DNA Extraction and Illumina Sequencing

Total genomic DNA was extracted from each specimen using QIAGEN DNeasy 96 Tissue Kits, following the manufacturer's protocol. Genomic libraries for mitochondrial genome skimming (Straub et al. 2012; Dodsworth 2015) were prepared for six individuals of the *Caiman yacare* using the Illumina Nextera XT DNA Library Preparation Kit, which includes blunt‐end repair, adapter ligation, adapter fill‐in, and indexing PCR (13 cycles), following the protocol developed by Meyer and Kircher (2010). Sequencing and library preparation were performed by NOVOGENE. Briefly, indexed libraries were pooled based on their relative concentrations to ensure equimolarity, and subjected to paired‐end sequencing (150 bp reads) on an Illumina NovaSeq lane. Mitogenomes were assembled de novo using MEGAHIT (Li et al. 2015), and annotated with MitoZ (Meng et al. 2019). Annotations were compared to the reference genomes available in GenBank for the *Caiman yacare* (captive individual from St. Augustine Alligator Farm Zoological Park, Accession number MN885913) and manually edited. The mitochondrial genome map was drawn using ORGDRAW (Greiner et al. 2019). The newly generated mitogenomes have been deposited in GenBank (Accession numbers PX092354‐PX092359). The number of distinct haplotypes and the nucleotide substitutions among them were estimated by reconstructing a haplotype network using the R package pegas (Paradis 2010).

### 2.4 Genetic Species Delimitation

To identify the phylogenetic position of the newly generated mitogenomes, publicly available mitochondrial genomes (Pan et al. 2021) and partial sequences of the mitochondrial cytochrome b gene (Farias et al. 2004; Hrbek et al. 2008; Vasconcelos et al. 2008; Venegas‐Anaya et al. 2008; Oaks 2011; Roberto et al. 2020; Amavet et al. 2023) were mined in NCBI GenBank and included in the analysis. An inventory of Molecular Operational Taxonomic Units (MOTUs), defined as diagnosable mitochondrial lineages, was conducted by applying five species delimitation methods to the complete dataset. The following algorithms were used: (1) Assemble Species by Automatic Partitioning (ASAP; Puillandre et al. 2021), available at https://bio.tools/asap‐assemble; (2) Poisson Tree Processes (PTP; Zhang et al. 2013), including the single‐threshold (sPTP) and (3) multiple‐threshold (mPTP) models; and (4) the Generalized Mixed Yule‐Coalescent model (GMYC; Fujisawa and Barraclough 2013), in both its single‐rate (sGMYC) and (5) multiple‐rate (mGMYC) versions, as implemented in the R package Splits v1.0‐19 within R v4.3.2 (R Core Team 2026). The final MOTU delimitation scheme was established based on a majority‐rule consensus across the five methods (Arida et al. 2021).

Both RESL and ABGD used DNA alignments as input. For the PTP analyses, a maximum likelihood (ML) tree was generated using IQ‐TREE v1.6.12 (Nguyen et al. 2015), applying the best‐fit substitution model (TVM + Γ) selected by ModelFinder (Kalyaanamoorthy et al. 2017) based on the Bayesian Information Criterion (BIC), available at https://iqtree.cibiv.univie.ac.at (Trifinopoulos et al. 2016). For GMYC analyses, the ultrametric and fully resolved tree required was reconstructed using a Bayesian approach implemented in BEAST v2.4.8 (Bouckaert et al. 2014). BEAST was run with three independent Markov chain Monte Carlo (MCMC) chains in order to reach ESS > 200, each consisting of 20 million generations, using a Yule pure‐birth tree prior, a relaxed log‐normal molecular clock and TVM + Γ substitution model. A substitution rate of 0.8% per million years (Myr) was applied for divergence time estimation, and corresponding to an average complete mitochondrial genome clock for vertebrates (Nabholz et al. 2009; Sholihah et al. 2021). Trees were sampled every 10,000 generations after an initial burn‐in of 5 million generations. Outputs from the three runs were combined using LogCombiner v2.4.8, and a maximum clade credibility tree with common ancestor heights was generated using TreeAnnotator v2.4.7 (Bouckaert et al. 2014). Prior to Bayesian inference, sequences were collapsed into haplotypes.

### 2.5 Phylogenetic Analyses

Once MOTUs were delimited, partial cytochrome b sequences and complete mitogenomes were used to reconstruct phylogenetic trees with the StarBEAST2 package (Ogilvie et al. 2017) in BEAST v2.4.8. As StarBEAST2 jointly estimates gene trees and species (MOTU) trees, MOTU assignments were based on the majority‐rule consensus from the lineage delimitation analyses. The analyses were performed using a single partition including the entire mitochondrial genome, an Uncorrelated Log‐Normal (UCLN) species tree prior, and a relaxed log‐normal molecular clock to account for rate variation among lineages. A GTR + Γ substitution model was applied as the TVM + Γ substitution model is not available in StarBEAST2. Three MCMC runs consisted of 20 million steps, with a substitution rate of 0.8% per million years (Myr). Independent runs were combined using LogCombiner v1.10.4 (Bouckaert et al. 2014). Maximum clade credibility trees for both gene and MOTU datasets, along with node age estimates and 95% highest posterior density (HPD) intervals, were summarized using TreeAnnotator v1.10.4 (Bouckaert et al. 2014).

Finally, a coalescent tree of *C. yacare* was reconstructed using the complete cytochrome b gene under a standard template in BEAST v2.4.8. The analysis included three independent Markov chain Monte Carlo (MCMC) runs, each consisting of 20 million generations, using a Yule pure‐birth tree prior, a relaxed log‐normal molecular clock, a substitution rate of 1.2% genetic divergence per million years, based on the calibration of mitochondrial protein‐coding gene divergence across the Isthmus of Panama in multiple species pairs of vertebrates and invertebrates (Knowlton et al. 1992; Bermingham et al. 1997), and a standard GTR + I + Γ substitution model. All substitution model parameters were jointly estimated along with divergence times and tree topology. Trees were sampled every 10,000 generations after a burn‐in period of 5 million generations. The outputs from the three runs were combined using LogCombiner v2.4.8, and a maximum clade credibility (MCC) tree with common ancestor heights was generated using TreeAnnotator v2.4.7.

## 3 Results and Discussion

**FIGURE 2:**
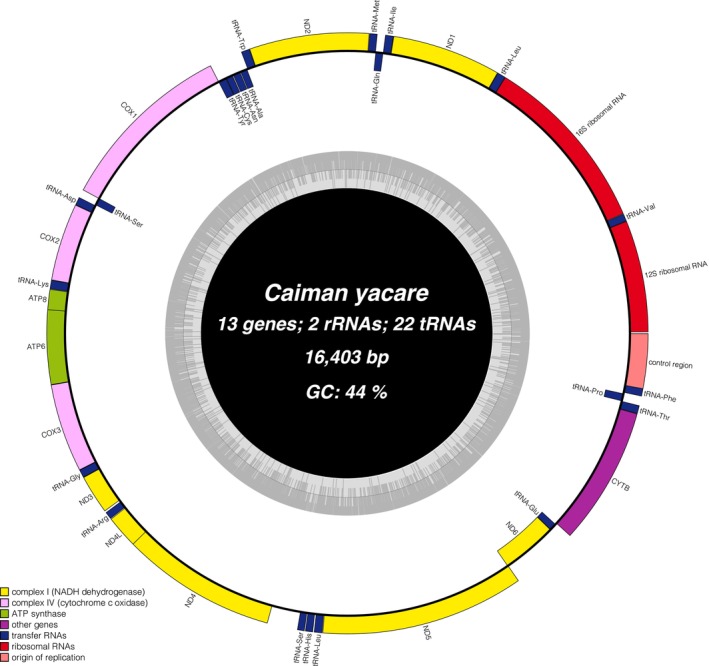
Genome map of *Caiman yacare* mitochondrial genome drawn using ORGDRAW (Greiner et al. 2019). Inner and outer genes are translated clockwise and counterclockwise, respectively. The inner circle represents the GC content.

**FIGURE 3:**
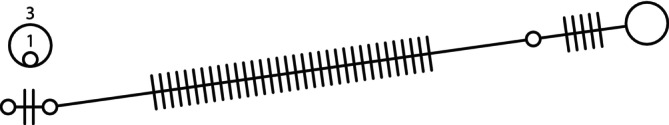
Haplotype network representing the evolutionary relationships among the four haplotypes detected in the six complete mitochondrial genomes sequenced in the present study.

A total of six high‐quality mitochondrial genomes were assembled, each with a minimum sequencing depth of 100× (File S1). The mitogenomes were 16,403 bp in length, and no insertions or deletions were detected. Each genome included two ribosomal RNA genes (982 bp and 1592 bp), 13 protein‐coding genes ranging from 293 to 1796 bp, and 22 tRNA genes ranging from 67 to 77 bp (Table 1, Figure 2). This gene arrangement is typical of vertebrate mitochondrial genomes (Boore 1999). A total of four haplotypes were identified among the six mitochondrial genomes, with 41 nucleotide substitutions reconstructed—36 of which occurred between two sets of closely related haplotypes (Figure 3).

**TABLE 1:** Composition of mitochondrial genome of the *Caiman yacare* including genes position, length and variability.

Product	Start	End	Length (bp)	Sens	Name
rRNA	11	998	987		12S rRNA
rRNA	1068	2659	1591		16S rRNA
tRNA	999	1066	67		tRNA‐Val
tRNA	2660	2734	74		tRNA‐Leu
tRNA	3699	3771	72		tRNA‐Ile
tRNA	3770	3840	70	Complement	tRNA‐Gln
tRNA	3840	3908	68		tRNA‐Met
tRNA	4968	5043	75		tRNA‐Trp
tRNA	5049	5114	65	Complement	tRNA‐Ala
tRNA	5118	5189	71	Complement	tRNA‐Asn
tRNA	5194	5259	65	Complement	tRNA‐Cys
tRNA	5260	5337	77	Complement	tRNA‐Tyr
tRNA	6901	6971	70	Complement	tRNA‐Ser
tRNA	6976	7048	72		tRNA‐Asp
tRNA	7737	7814	77		tRNA‐Lys
tRNA	9434	9505	71		tRNA‐Gly
tRNA	9868	9935	67		tRNA‐Arg
tRNA	11864	11928	64		tRNA‐Ser
tRNA	11934	12001	67		tRNA‐His
tRNA	12011	12083	72		tRNA‐Leu
tRNA	14397	14465	68	Complement	tRNA‐Glu
tRNA	15697	15770	73		tRNA‐Thr
tRNA	15771	15842	71	Complement	tRNA‐Pro
tRNA	15852	15921	69		tRNA‐Phe
Misc	15917	16403	486		Control region
CDS	2735	3698	963		NADH dehydrogenase subunit 1 (ND1)
CDS	3910	4967	1057		NADH dehydrogenase subunit 2 (ND2)
CDS	5334	6905	1571		Cytochrome c oxidase subunit I (COXI)
CDS	7049	7736	687		Cytochrome c oxidase subunit II (COXII)
CDS	7816	7998	182		ATPase subunit 8 (ATPase8)
CDS	7968	8651	683		ATPase subunit 6 (APTase6)
CDS	8651	9434	783		Cytochrome c oxidase subunit III (COXIII)
CDS	9506	9853	347		NADH dehydrogenase subunit 3 (ND3)
CDS	9939	10232	293		NADH dehydrogenase subunit 4 L (ND4L)
CDS	10226	11599	1373		NADH dehydrogenase subunit 4 (ND4)
CDS	12086	13882	1796		NADH dehydrogenase subunit 5 (ND5)
CDS	13875	14396	521	Complement	NADH dehydrogenase subunit 6 (ND6)
CDS	14475	15696	1221		Cytochrome b (cytb)

**FIGURE 4:**
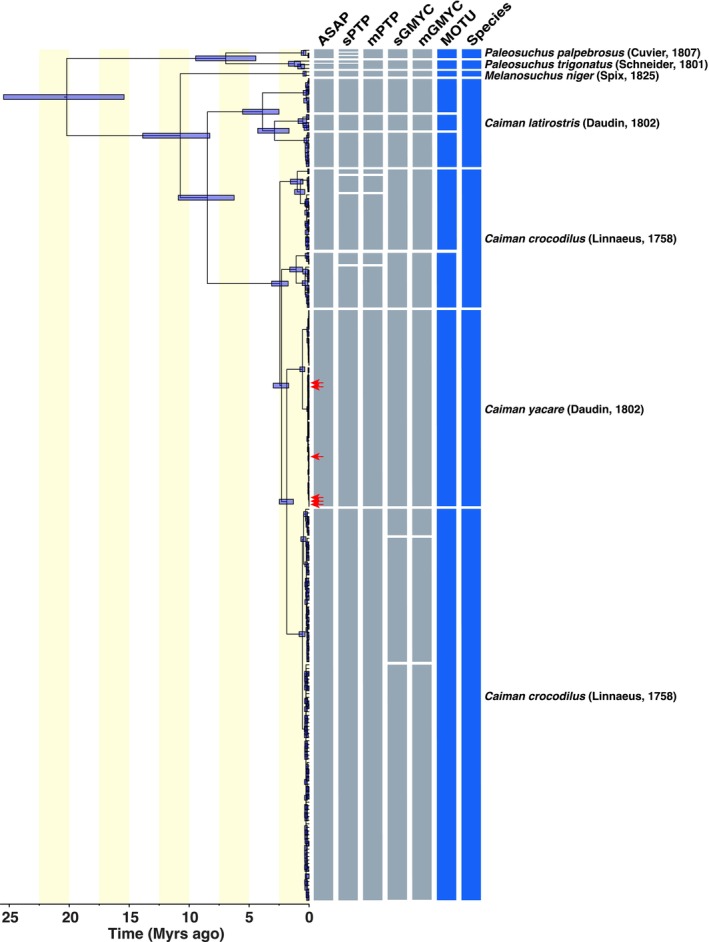
Mitochondrial gene tree of Crocodilians inferred using complete mitochondrial genomes and partial sequences of the mitochondrial gene coding for the Cytochrome b, including species delimitation schemes (ASAP, GMYC, PTP) and the majority‐rule consensus scheme (MOTU).

With the objectives to confirm the identity of the Tuichi population and to examine the maternal genetic structure of this population in comparison to lowland populations, a total of 14 mitochondrial genomes from the genera *Paleosuchus*, *Melanosuchus*, and *Caiman* were downloaded from GenBank, along with 216 complete cytochrome b gene sequences (Table S1 and File S1). A total of seven MOTUs were delimited within the genus *Caiman* based on the consensus of the five species delimitation algorithms (Figure 4; Table S1). *Caiman crocodilus* and *C. latirostris* each encompass three MOTUs, with *C. crocodilus* mitochondrial lineages being polyphyletic, while *C. yacare* consists of a single MOTU. The six newly generated mitogenomes cluster within the *C. yacare* MOTU, confirming that the Tuichi population belongs to *C. yacare* .

Combined with the six newly generated mitogenomes, a total of 236 mitochondrial sequences were used to reconstruct a Bayesian gene tree using the SpeciesTreeUCLN model (Figure 4). The genus *Caiman* was recovered as monophyletic in our phylogenetic reconstruction, with its most recent common ancestor (MRCA) dated between 7 and 11 million years ago (Table 2). This estimated age of the MRCA of *Caiman* falls within the lower range of published estimates, overlapping with Oaks (2011), who date the MRCA to 7–11 Myr, and approaching the estimate of Pan et al. (2021), who inferred an age of 13–15 Myr based on mitochondrial genomes. Our estimate largely differs, however, from those from Amavet et al. (2023) and Roberto et al. (2020), who dated the origin of Caiman at 47 and 19–42 Myr, respectively (Table 2). These substantial differences likely stem from the calibration strategies employed, as Amavet et al. (2023) and Roberto et al. (2020) calibrated the MRCA of Alligatorinae based on *Novajosuchus mooki*, the oldest known fossil attributed to the subfamily, and possibly belonging to the stem group leading to modern Alligatorinae. Similarly, the 0.8% divergence applied here may overestimate mitochondrial rates for Alligatorinae, since lineages with longer generation times typically exhibit slower substitution rates (Nabholz et al. 2016). The age estimates reported here align with the period of establishment of the major tributaries of the Amazon watershed, which occurred within the last 10 Myr (Lundberg et al. 1998; Hubert and Renno 2006; Albert and Reis 2011). These results underscore the need for further investigations, which lie beyond the scope of the present study.

**TABLE 2:** Age estimates for several MRCAs within Alligatorinae based on previously published studies and the present study. The estimates presented here for the MRCA of *Caiman* + *Melanosuchus* + *Paleosuchus*, *Caiman* + *Melanosuchus*, and *Caiman* are based on a substitution rate of 0.8% per million years.

TMRCA	Oaks (2011)		Pan et al. (2021)		Amavet et al. (2023)		Roberto et al. (2020)		Present study	
	Mean	95% HPD	Mean	95% HPD	Mean	95% HPD	Mean	95% HPD	Mean	95% HPD
*Caiman* + *Melanosuchus* + *Paleosuchus*	25.37a	20.14–28.81	36.18	34.26–38.16			61.93	42.9–91.0	20.20	15.43–25.47
	29.01b	24.85–32.55								
*Caiman* + *Melanosuchus*	12.43a	10.31–14.68	17.4	16.36–18.41			38.75	25.9–57.8	10.75	8.28–13.87
	13.1b	10.60–16.87								
*Caiman*	8.34a	6.69–9.81	13.81	12.93–14.74	47	—	27.73	18.5–41.98	8.47	6.25–10.91
	8.67b	7.06–10.89								

**FIGURE 5:**
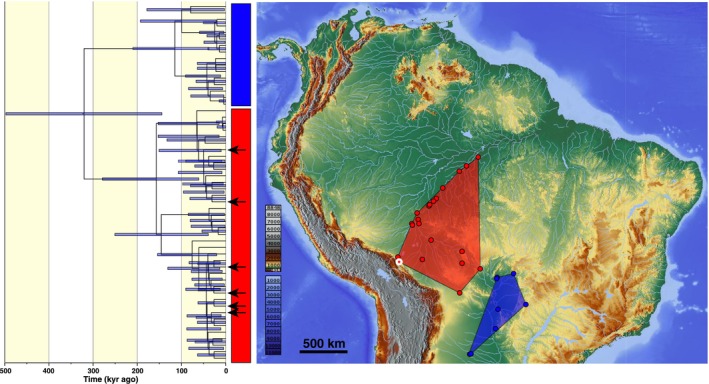
Distribution of the specimens corresponding to the two phylogroups within the *Caiman yacare* , including the specimens used to produce the six new mitochondrial genomes.

The gene tree of *C. yacare* forms a cluster of closely related haplotypes, consistently delimited as a single MOTU across all methods. However, the cytochrome b coalescent gene tree of *C. yacare* reveals two phylogroups with distinct geographic distributions (Figure 5). The MRCA of all cytochrome b haplotypes of *C. yacare* is dated between 144,400 and 497,400 years ago, while the MRCA of the Madeira phylogroup (Figure 5, red circle) is dated between 60,900 and 279,100 years ago. All six newly generated mitochondrial genomes fall within the Madeira phylogroup, indicating close affinities with lowland populations in the Madeira.

## Author Contributions

**Guido Miranda:** conceptualization (equal), data curation (equal), formal analysis (equal), funding acquisition (equal), investigation (lead), project administration (lead), supervision (equal), validation (equal), writing – original draft (equal). **Ninón Ríos:** data curation (equal), investigation (equal), project administration (equal), validation (equal), visualization (equal). **Nicolas Hubert:** conceptualization (equal), data curation (equal), formal analysis (lead), funding acquisition (equal), investigation (equal), methodology (lead), project administration (equal), supervision (equal), writing – original draft (equal).

## Funding

This study was funded by IRD through recurrent funding.

## Conflicts of Interest

The authors declare no conflicts of interest.

## Supporting information


**Data S1:** Mitochondrial DNA sequence alignment including 20 mitochondrial genomes (i.e., six newly generated mitogenomes presented here and 14 downloaded from NCBI GenBank) and 216 complete cytochrome b gene sequences downloaded from NCBI GenBank.
**Table S1:** Origin of the 236 mitochondrial sequences analyzed in the present study.

## Data Availability

All the required data are uploaded as Supporting Information. Mitogenomes sequences were deposited in GenBank (Accession number PX092354‐PX092359).
